# Development and Validation of a Nomogram for Predicting Drug-Induced Acute Kidney Injury in Hospitalized Patients: A Case-Control Study Based on Propensity-Score Matching

**DOI:** 10.3389/fphar.2021.657853

**Published:** 2021-06-14

**Authors:** Chengxuan Yu, Daihong Guo, Chong Yao, Yu Zhu, Siyuan Liu, Xianghao Kong

**Affiliations:** ^1^ Pharmacy Department, Medical Security Center, Chinese PLA General Hospital, Beijing, China; ^2^ Graduate School, Chinese PLA General Hospital, Beijing, China; ^3^ College of Pharmacy, Chongqing Medical University, Chongqing, China

**Keywords:** adverse drug reaction, drug-induced acute kidney injury, active surveillance, informatization, nomogram, predictive model

## Abstract

**Background:** Drug-induced acute kidney injury (D-AKI) is associated with increased mortality and longer hospital stays. This study aims to establish a nomogram to predict the occurrence of D-AKI in hospitalized patients in a multi-drug environment.

**Methods:** A single center retrospective study among adult hospitalized patients was conducted from July 2019 to September 2019 based on the Adverse Drug Events Active Surveillance and Assessment System-2 developed by our hospital. According to the propensity score matching algorithm, four controls per case were matched to eliminate the confounding bias caused by individual baseline variables. The predictors for D-AKI were obtained by logistic regression equation and used to establish the nomogram.

**Results:** Among 51,772 hospitalized patients, 332 were diagnosed with D-AKI. After matching, 288 pairs and 1,440 patients were included in the study, including 1,005 cases in the development group and 435 cases in the validation group. Six variables were independent predictors for D-AKI: alcohol abuse, the concurrent use of nonsteroidal anti-inflammatory drugs or diuretics, chronic kidney disease, lower baseline red blood cell count and neutrophil count ≥7 × 10^9^/L. The area under the curve (AUC) of the prediction model in the development group and validation group were 0.787 (95%CI, 0.752–0.823) and 0.788 (95%CI, 0.736–0.840), respectively. The GiViTI calibration belts showed that the model had a good prediction accuracy for the occurrence of D-AKI (*p* > 0.05).

**Conclusion:** This nomogram can help identify patients at high risk of D-AKI, which was useful in preventing the progression of D-AKI and treating it in the early stages.

## Introduction

Acute kidney injury (AKI) is one of the most serious complications of hospitalized patients, and is associated with increased risk of death and higher costs (Ruiz-Criado et al., 2015; Sykes et al., 2018). Drugs have become the third to fifth leading cause of AKI in hospitalized patients (Kane-Gill and Goldstein, 2015). Drug-induced acute kidney injury (D-AKI) is defined as kidney injury caused by drugs or their metabolites within 7 days after the use of one or more drugs (Awdishu and Mehta, 2017). D-AKI is increasingly recognized as a relatively common adverse drug reaction (ADR) in clinical practice. In the United States, 18%–27% of hospital-acquired AKI cases are caused by drugs (Taber and Pasko, 2008; Pierson-Marchandise et al., 2017). In China, the proportion of D-AKI in all AKI cases has risen from 26.5% to 42.9% in recent years (Che et al., 2009; Xu et al., 2015). A national multi-center AKI survey study has shown that 71.6% of AKI patients in Chinese hospitalized patients had a history of potential nephrotoxic drug use before or during kidney injury (Yang et al., 2015).

Prevention is the key to avoiding the heavy burden of mortality and morbidity caused by AKI (Li et al., 2013). Therefore, early detection of patients at risk of D-AKI will help to use medical resources more effectively, formulate D-AKI prevention strategies, and improve patient safety. Although the increase in serum creatinine (Scr) is the cornerstone of the diagnosis of AKI, its early recognition is hindered by many factors and is less accurate and timely (Kashani et al., 2018). AKI biomarkers, such as cystatin C and neutrophil gelatinase-associated lipocalin, may improve risk assessment (Markwardt et al., 2017), but these tests are expensive and far away from extensive clinical applications. Thus, it is particularly important to develop a clinically convenient, accurate and efficient D-AKI risk prediction model. Currently, nomograms are widely used to predict D-AKI because of their intuitiveness and easy-to-understand character. However, the construction of D-AKI risk prediction models in recent studies were mostly based on specific patients (Mehran et al., 2004; Zhou et al., 2018; Patidar et al., 2019). Nomograms are rarely used to predict the risk of D-AKI in the general hospital population in a multi-drug environment.

Therefore, based on the Adverse Drug Events Active Surveillance and Assessment System-2 (ADE-ASAS-2) developed by our hospital, we carried out a case-control study with propensity score matching to explore the clinical characteristics of patients with D-AKI and constructed a nomogram to predict the risk of D-AKI in hospitalized patients.

## Materials and Methods

### Study Design and Patient Selection

This single-center, retrospective, propensity-score matched case-control study was performed in a group of hospitalized patients over the age of 18. Eligible patients included those who developed AKI after treatment with nephrotoxic drugs in our hospital from June 30, 2019 to September 30, 2019.

All patient data including medical records and examination information were obtained from the Hospital Information System (HIS). The ADE-ASAS-2 was based on trigger technology and text recognition technology, which can be connected to the HIS to extract patient information. Detailed descriptions of the ADE-ASAS-2 and its applications have been described in our previous study (Chen et al., 2020; Yu et al., 2020). Once the monitoring indicators trigger the inclusion criteria, the system could preliminarily determine whether a patient has developed AKI and indicate early warning signals. Since the monitoring plan was carried out in the full prescription mode, and without setting up monitoring drugs, drugs with a temporal relationship to SCr elevation could be captured as D-AKI early warning signals. An alarm case may include multiple early warning signals. Then, two clinical pharmacists conducted back-to-back evaluations to confirm the alarm results, and cases with inconsistent assessment results were referred to an expert for final judgment to determine whether D-AKI had occurred.

We defined AKI on the basis of SCr definition criteria of 2012 Kidney Disease: Improving Global Outcomes (KDIGO) Clinical Practice Guidelines for Acute Kidney Injury (Kellum et al., 2012), the inclusion criteria were as follows: 1) age ≥18 years old; 2) a full prescription model (temporary prescriptions and long-term prescriptions); 3) an increase in SCr by at least 0.3 mg/dL within 48 h or an increase in SCr to at least 1.5 times higher than baseline within the prior 7 days. Patients with baseline SCr >5 mg/dL or no baseline SCr data were automatically excluded (*n* = 3824). The other exclusion criteria were as follows: 1) patients with stage 5 chronic kidney disease (CKD) (*n* = 61); 2) patients with absent laboratory indexes before or after medication administration (*n* = 97); 3) patients undergoing dialysis or who underwent nephrectomy or kidney transplantation (*n* = 38); 4) patients with incomplete clinical records (*n* = 133). The Naranjo Adverse Drug Reaction Probability Scale (Naranjo Scale) was used as a causality assessment tool (Naranjo et al., 1981). The ADR was assigned to a probability category from the total score as follows: definite ≥9, probable 5 to 8, possible 1 to 4, and doubtful ≤0. Patients with scores ≥1 were defined as D-AKI.

Controls were defined as patients treated with similar nephrotoxic drugs who did not develop AKI. To minimize confounding bias due to demographic characteristics, length of hospital stays, and duration of suspected drug exposure, we performed a 1:4 propensity core matching between the D-AKI and non-D-AKI groups to make the groups more comparable.

### Data Collection and Definitions

Patient data were monitored and extracted from the HIS through the ADE-ASAS-2. We collected the following variables. Patient characteristics included age, gender, body mass index (BMI), smoking history, alcohol abuse, hospital stay, suspected drug duration and surgical history. Comorbidities included diabetes mellitus (DM) (defined as at least 2 fasting blood glucose measurements >7 mmol/L or use of antidiabetic agents), hypertension (defined as previous diagnosis of hypertension, previous use of antihypertensive medications, or at least 2 separate measurements of systolic pressure >140 mmHg and/or a diastolic pressure >90 mmHg during hospitalization), cardiovascular disease (including diagnoses of congestive heart failure, myocardial infarction, unstable angina pectoris, etc.), anemia (defined as a baseline hemoglobin value below 130 g/L in men and 120 g/L in women) and CKD [defined as having an (estimated glomerular filtration rate) eGFR <60 mL/min/1.73 m^2^ for 3 months with or without kidney damage or CKD explicitly mentioned in the admission diagnosis]. Concomitant use of drugs included angiotensin-converting enzyme inhibitors (ACEIs), angiotensin receptor blockers (ARBs), nonsteroidal anti-inflammatory drugs (NSAIDs), diuretics and vancomycin. Laboratory data included SCr, uric acid (UA), hemoglobin (HB), red blood cell (RBC) count, white blood cell (WBC) count, platelet (PLT) count. Secondary data, such as the eGFR, were calculated as needed. We used the Chronic Kidney Disease Epidemiology Collaboration creatinine equation (CKD-EPI) to calculate eGFR, because it is more accurate than the Modification of Diet in Renal Disease (MDRD) formula, as recommended by clinical practice guidelines (Kellum et al., 2012; Levey et al., 2009). The SCr baseline was defined as the last laboratory measurement between 7 days before and 2 h after the suspected drug administration. Other laboratory values were collected at the time of the most recent laboratory measurement prior to suspected drug administration.

### Statistical Analysis

SPSS statistical software (version 25.0, SPSS, IBM Corporation, United States), STATA software (version 16.0, Stata Corporation LLC, United States) and R software (version 4.0.3, the R Core Team; United States) were used to perform the statistical analysis and model development. For baseline characteristics, All data did not conform to a normal distribution, so quantitative data were expressed as median and interquartile range (IQR) and compared by Mann-Whitney U test. Qualitative data were expressed as numbers and percentages and compared by the χ2 test or Fisher’s exact test.

To make baseline characteristics similar between groups, the propensity core matching method was used to match D-AKI patients and non-D-AKI patients using the same suspected drugs. Matching with a ratio of 1:4 was performed (caliper value is 0.05) using the “psmatch2 package” of STATA software. Propensity scores were estimated by age, gender, BMI, hospital stay and suspected drug duration.

To identify the independent predictors for D-AKI, binary logistic regression analysis was used. Variables that differed significantly in univariate analysis were included in a multivariate logistic regression model (*p* < 0.05 was used for entry, and *p* > 0.10 for removal) using the Forward: LR mode. Estimates of odds ratios (ORs) and 95% confidence intervals (CIs) for predictors were computed. A nomogram was constructed according to the results of multivariable logistic regression using the “rms package” in R software. Referring to the grouping ratios in previous studies (Zhou et al., 2018; Wang Q. et al., 2019), the ratio of the development and validation groups was divided into 7:3 in this study.

The discrimination of the model was evaluated by the receiver operating characteristic (ROC) curves constructed by the “pROC package” in R software, and area under the curve (AUC) was calculated. The calibration of the model was evaluated by the GiViTI calibration belts, which were implemented by the “givitiR package” in R software. In the GiViTI calibration test, the prediction model was considered poorly calibrated when *p* < 0.05; conversely, when *p* > 0.05, it was not sufficient to demonstrate a lack of model fit. A value of *p* < 0.05 was considered significant. All the reported *p*-values were 2-sided.

## Results

### Clinical Characteristics of the Patients

**FIGURE 1:**
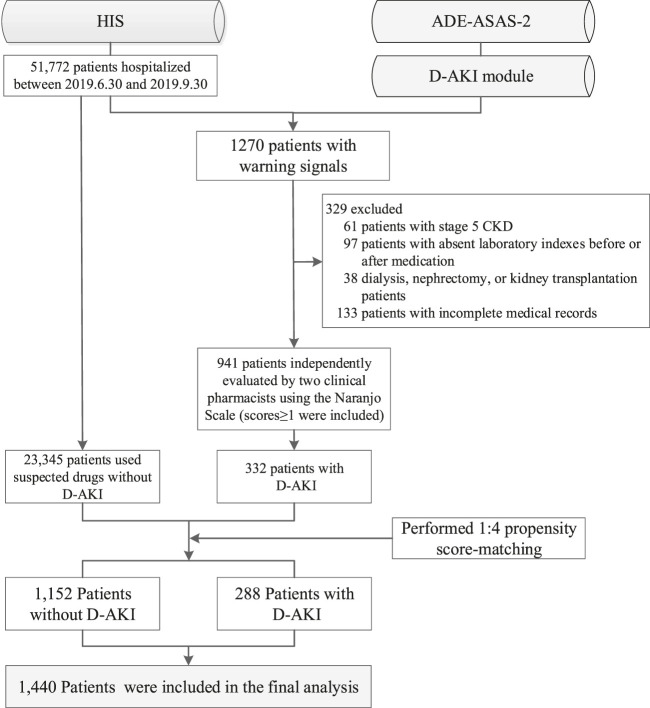
Flow diagram of study population enrollment. HIS, hospital information system; ADE-ASAS-2, Adverse Drug Events Active Surveillance and Assessment System-2; D-AKI, drug-induced acute kidney injury; CKD, chronic kidney disease.

Of the patients admitted from June 30, 2019 to September 30, 2019, a total of 51,772 were monitored by ADE-ASAS-2, 3,290 (6.35%) were automatically excluded by ADE-ASAS-2, and 1,270 (2.45%) were indicated warning signals. Among these patients, 329 (25.91%) were excluded according to our exclusion criteria. After independent evaluation by two clinical pharmacists using the Naranjo Scale to exclude 609 patients with no causal relationship to the drug, we finally identified that 332 (26.14%) patients were diagnosed with D-AKI. The overall incidence of D-AKI in hospitalized Chinese patients (aged ≥18) was 0.64%. Among the hospitalized patients during the study period, 23,345 patients used the same suspected drugs but did not develop D-AKI. After propensity score matching, 288 cases and 1,152 controls were finally paired. The selection process is summarized in Figure 1. Baseline demographic and clinical characteristics before and after matching are described in Table 1. After matching, there was no significant difference between groups for each matching variable (*p* > 0.05). A total of 66 drugs were administered in the 332 D-AKI cases. This study finally included 1,440 patients for analysis, randomized in a 7:3 ratio into the development group (*n* = 1005) and a validation group (*n* = 435). The baseline clinical characteristics of the development and validation groups are shown in Table 2.

**TABLE 1:** Baseline characteristics of patients before and after propensity score matched analysis.

Variables	Before matched (*n* = 23677)			After matched (*n* = 1440)		
	D-AKI (*N* = 332)	No D-AKI (*n* = 23345)	*p* value	D-AKI (*n* = 288)	No D-AKI (*n* = 1152)	*p* value
Median age, years (IQR)	65 (53–75)	56 (44–66)	<0.001	64 (53–73)	65 (54–74)	0.497
Males, n (%)	214 (64.5)	12913 (55.3)	0.001	186 (64.6)	764 (66.3)	0.578
Median BMI, kg/m^2^ (IQR)	23.3 (20.8–26.0)	24.0 (21.7–26.6)	<0.001	23.4 (21.1–26.1)	23.6 (21.0–25.8)	0.950
Median hospital stay, days (IQR)	20 (12–34)	9 (6–14)	<0.001	16 (10–24)	15 (19–24)	0.196
Median suspected drug duration, days (IQR)	4 (2–6)	3 (1–6)	0.022	4 (2–6)	3 (1–7)	0.356

IQR, interquartile range, BMI, body mass index.

**TABLE 2:** Baseline characteristics in the development and validation groups.

Variables	Development group (*n* = 1005)			Validation group (*n* = 435)		
	D-AKI (*n* = 201)	Non D-AKI (*n* = 804)	*p* value	D-AKI (*n* = 87)	Non D-AKI (*n* = 348)	*p* value
**Demographic data**						
Median age, years (IQR)	64 (53–73)	65 (54–74)	0.241	65 (53–77)	65 (52–75)	0.616
Males, n (%)	135 (67.2)	541 (67.3)	0.973	51 (58.6)	223 (64.1)	0.345
Median BMI, kg/m^2^ (IQR)	23.8 (21.8–26.8)	24.1 (21.6–26.0)	0.656	22.0 (20.4–24.8)	22.2 (20.2–25.1)	0.609
Smoking history, n (%)	92 (45.8)	277 (34.5)	0.003	28 (32.2)	93 (26.7)	0.309
Alcohol abuse, n (%)	80 (39.8)	258 (32.1)	0.038	26 (29.9)	79 (22.7)	0.161
Median hospital stay, days (IQR)	16 (10–22)	14 (8–22)	0.175	16 (12–33)	17 (11–28)	0.605
Median suspected drug duration, days (IQR)	3 (2–6)	3 (1–6)	0.212	5 (2–9)	5 (3–8)	0.986
Surgical history, n (%)	54 (26.9)	241 (30.0)	0.387	38 (43.7)	86 (24.7)	<0.001
**Concomitant use of drugs**						
ACEIs, n (%)	10 (5.0)	24 (3.0)	0.163	5 (5.7)	22 (6.3)	0.842
ARBs, n (%)	23 (11.4)	49 (6.1)	0.009	21 (24.1)	26 (7.5)	<0.001
NSAIDs, n (%)	85 (42.3)	172 (21.4)	<0.001	31 (35.6)	67 (19.3)	0.001
Diuretics, n (%)	96 (47.8)	120 (14.9)	<0.001	47 (54.0)	54 (15.5)	<0.001
Vancomycin, n (%)	6 (3.0)	35 (4.4)	0.380	7 (8.0)	12 (3.4)	0.061
**Comorbidities**						
Diabetes mellitus, n (%)	46 (22.9)	127 (15.8)	0.017	27 (31.0)	59 (17.0)	0.003
Hypertension, n (%)	86 (42.8)	309 (38.4)	0.258	42 (48.3)	131 (37.6)	0.070
Cardiovascular disease, n (%)	57 (28.4)	139 (17.3)	<0.001	39 (44.8)	50 (14.4)	<0.001
Anemia, n (%)	40 (19.9)	83 (10.3)	<0.001	51 (58.6)	111 (31.9)	<0.001
CKD, n (%)	25 (12.4)	37 (4.6)	<0.001	16 (18.4)	28 (8.0)	0.004
**Laboratory measurements**						
Median baseline SCr, mg/dL (IQR)	0.95 (0.69–1.45)	0.83 (0.68–1.01)	<0.001	0.94 (0.69–1.37)	0.82 (0.66–1.01)	0.007
Median UA, mmol/L (IQR)	310.2 (223.4–384.1)	282.0 (215.0–354.4)	0.017	257.0 (197.6–416.3)	270.8 (194.9–356.3)	0.693
Median HB, g/L (IQR)	106 (90–127)	120 (102–135)	<0.001	257.0 (197.6–416.3)	270.75 (194.90–356.28)	0.002
Median RBC count, ×10^12^/L (IQR)	3.47 (2.96–4.18)	3.93 (3.43–4.44)	<0.001	3.31 (2.66–3.84)	3.67 (4.52–9.69)	0.005
Median WBC count, ×10^9^/L (IQR)	10.30 (7.10–13.35)	7.83 (5.55–10.91)	<0.001	9.60 (6.07–14.95)	6.98 (4.52–9.69)	<0.001
Median neutrophil count, ×10^9^/L (IQR)	8.62 (5.22–11.64)	5.59 (3.38–8.97)	<0.001	7.25 (4.35–13.11)	5.01 (2.86–7.64)	<0.001
Neutrophil count≥7×10^9^/L, n (%)	123 (61.2)	307 (38.2)	<0.001	45 (51.7)	104 (29.9)	<0.001
Median lymphocyte count, ×10^9^/L (IQR)	0.94 (0.60–1.51)	1.18 (0.74–1.72)	<0.001	0.82 (0.50–1.24)	0.41 (0.26–0.58)	0.013
Median PLT count, ×10^9^/L (IQR)	176 (121–245)	197 (146–247)	0.010	141 (78–198)	176 (111–230)	0.011
Median baseline eGFR, ml/min/1.73 m^2^ (IQR)	74.88 (49.54–97.04)	89.56 (71.11–101.61)	<0.001	74.66 (47.00–98.00)	91.66 (69.01–114.77)	0.001
Median NLR (IQR)	8.80 (4.25–15.87)	4.79 (2.28–10.16)	<0.001	10.39 (5.75–18.12)	4.97 (2.25–11.51)	<0.001
Median PLR (IQR)	175.62 (105.28–292.38)	155.78 (112.19–251.34)	0.213	166.28 (91.89–302.49)	158.32 (101.82–245.37)	0.520

IQR, interquartile range, BMI, body mass index; ACEIs, angiotensin-converting enzyme inhibitors; ARBs, angiotensin receptor blockers; NSAIDs, nonsteroidal anti-inflammatory drugs; CKD, chronic kidney disease; SCr, serum creatinine; UA, serum uric acid; HB, hemoglobin; RBC, red blood cell; WBC, white blood cell; eGFR, estimated glomerular filtration rate; NLR, neutrophil/lymphocyte ratio; PLR, platelet/lymphocyte ratio.

### Predictors of Drug-Induced Acute Kidney Injury

To confirm the predictors of D-AKI, univariate and multivariate logistic regression were performed. The potential predictors (*p* < 0.05 in the univariable analysis) were accepted for multivariate logistic regression. In the final regression model, the following variables were included: smoking history, alcohol abuse, ARBs, NSAIDS, diuretics, diabetes mellitus, cardiovascular disease, anemia, CKD, baseline SCr, baseline eGFR, baseline RBC count, neutrophil count ≥7 × 10^9^/L and NLR. In multivariate analysis, alcohol abuse (OR = 1.68, 95% CI: 1.17–2.41, *p* = 0.005), use of NSAIDs (OR = 2.56, 95% CI: 1.77–3.70, *p* < 0.001), use of diuretics (OR = 3.87, 95% CI: 2.67–5.62, *p* < 0.001), CKD (OR = 2.76, 95% CI: 1.51–5.04, *p* = 0.001), baseline RBC (OR = 0.61, 95% CI: 0.48–0.77, *p* < 0.001) and neutrophil count ≥7 × 10^9^/L (OR = 2.87, 95% CI: 2.02–4.09, *p* < 0.001) were independently associated with D-AKI. Table 3 shows the result of univariate and multivariate logistic regression analyses of 1,005 patients in the development group.

**TABLE 3:** Univariate and multivariate analysis of predictors for D-AKI.

Variables	Univariate		Multivariate	
	Odds ratio (95% CI)	*p* value	Odds ratio (95% CI)	*p* value
Smoking history	1.61 (1.17–2.20)	0.003		
Alcohol abuse	1.40 (1.02–1.93)	0.039	1.68 (1.17–2.41)	0.005
ARBs	1.99 (1.18–3.35)	0.010		
NSAIDs	2.69 (1.94–3.73)	<0.001	2.56 (1.77–3.70)	<0.001
Diuretics	5.21 (3.71–7.31)	<0.001	3.87 (2.67–5.62)	<0.001
Diabetes mellitus	1.58 (1.08–2.31)	0.018		
Cardiovascular disease	1.89 (1.33–2.71)	<0.001		
Anemia	2.16 (1.43–3.27)	<0.001		
CKD	2.95 (1.73–5.02)	<0.001	2.76 (1.51–5.04)	0.001
Baseline SCr	1.23 (1.06–1.43)	0.007		
Baseline eGFR	1.00 (0.98–1.00)	<0.001		
Baseline RBC	0.53 (0.43–0.65)	<0.001	0.61 (0.48–0.77)	<0.001
Neutrophil count ≥7 × 10^9^/L	2.55 (1.86–3.51)	<0.001	2.87 (2.02–4.09)	<0.001
NLR	1.02 (1.01–1.04)	<0.001		

D-AKI, drug-induced acute kidney injury; CI, confidence interval; ARBs, angiotensin receptor blockers; NSAIDs, nonsteroidal anti-inflammatory drugs; CKD, chronic kidney disease; SCr, serum creatinine; eGFR, estimated glomerular filtration rate; RBC, red blood cell; NLR, neutrophil/lymphocyte ratio.

### Development and Validation of Nomogram

**FIGURE 2:**
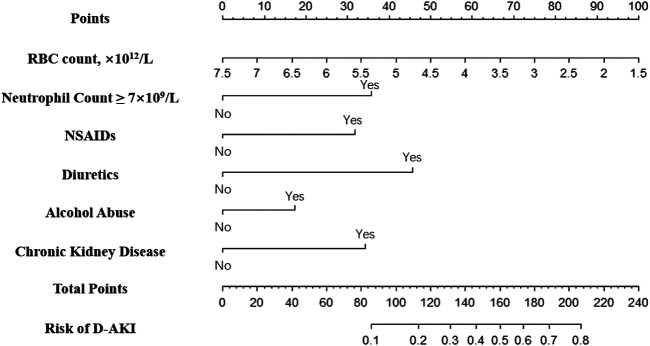
Nomogram for the prediction of D-AKI.

A nomogram was constructed based on independent predictors from multivariate logistics regression analysis for the prediction of D-AKI (Figure 2). In this nomogram, baseline RBC is a continuous variable, and other predictors are categorical variables, including neutrophil count ≥7 × 10^9^/L, use of NSAIDs, use of diuretics, alcohol abuse, and previous CKD. The nomogram has nine rows, with the first row being the point assignment of predictors and the second to seventh rows being predictors of D-AKI. The probability of AKI occurrence of D-AKI is predicted by matching the sum of the total scores (eighth row) with the scores on the total score table (ninth row).

**FIGURE 3:**
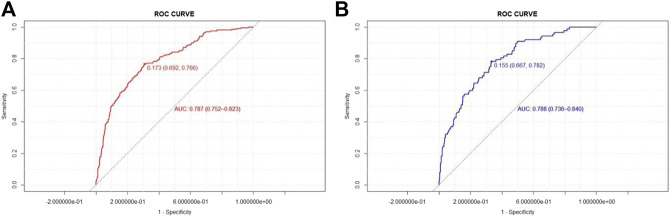
The ROC curve in the development **(A)** and validation **(B)** groups.

**FIGURE 4:**
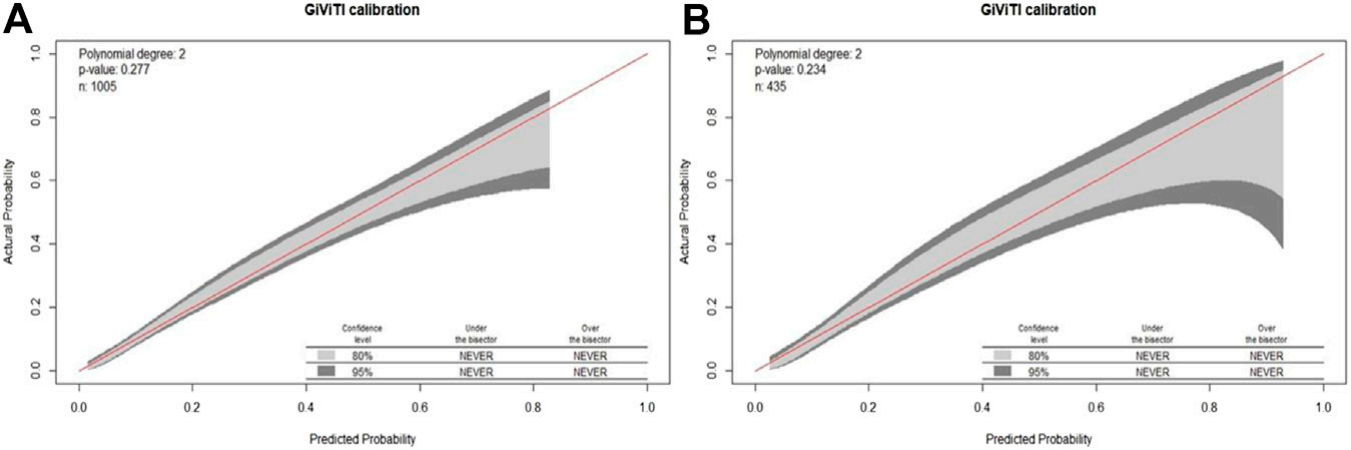
The GiViTI calibration belt in the development **(A)** and validation **(B)** groups.

To evaluate the model discrimination and reduce the overfitting bias, internal validation was performed in the validation group, Figure 3 shows the receiver operating characteristic (ROC) curves in the development and validation groups. In our study, the area under the curve (AUC) was 0.787 (95% CI 0.752–0.823) for the development group and 0.788 (95% CI 0.736–0.840) for the validation group. The nomogram model also indicated good calibration according to GiViTI calibration belts. The risk of D-AKI estimated by the nomogram was in good agreement with the actual observation results of D-AKI, with *p* values of 0.277 and 0.234 for the development and validation groups, respectively (Figure 4).

## Discussion

This study found that the total incidence of D-AKI in our adult inpatient population was about 0.64%. At present, there is still a lack of accurate incidence of D-AKI in all hospitalized populations. Previous reports in the literature have shown that the incidence of AKI in inpatients ranges from 0.7 to 77% depending on the definition and study population (James et al., 2010; Han et al., 2013), with a drug-related rate of 25% (Sales and Foresto, 1992). Therefore, the comprehensive incidence of D-AKI that we obtained is still within a reasonable range. Possible reasons for the lower incidence than in general studies include the fact that the study population was the entire hospitalized population and may have included individuals who were not exposed to nephrotoxic drugs, such as those who underwent health screening. In addition, the impact of the number of patients using low nephrotoxic drugs during hospitalization cannot be ignored, which also lowered the incidence rate.

Currently, there is no nomogram for predicting D-AKI risk based on the general Chinese patient population to accurately assess the risk of D-AKI in Chinese hospitalized patients in a multi-drug environment. Therefore, this research was dedicated to developing a simple and easy-to-use D-AKI prediction nomogram for adult inpatients to help clinicians to more accurately identify patients with potential D-AKI risk.

In clinical practice, clinicians can use this predictive model to assess the risk of D-AKI occurrence in newly admitted patients as needed and to initially classify patients based on this risk. Once high-risk patients are identified, urine output and serum creatinine must be diligently monitored (Rewa and Bagshaw, 2014). In addition, attempts should be made to manage potential risk factors, including discontinuing all nephrotoxic medications when possible, ensuring adequate volume status, monitoring serum creatinine, monitoring urine output, hemodynamic monitoring, and avoiding hyperglycemia (Kellum et al., 2012; Rewa and Bagshaw, 2014).

The D-AKI risk prediction nomogram was developed by the development group and verified in the validation group. Previous research experience has shown that an AUC value greater than 0.7 indicates good predictive performance of the model (Wang Q. et al., 2019; Bell et al., 2020). In our model, the AUC values for the development and validation groups were 0.787 (95%CI: 0.752–0.823) and 0.788 (95%CI: 0.736–0.840), respectively. The calibration of the model was verified by the GiViTI calibration belt. The *p* values were greater than 0.05 in both the development and validation groups. The above shows that our model has a good ability to distinguish D-AKI patients from non-D-AKI patients without overestimating or underestimating the risk of occurrence.

The nomogram model predicts D-AKI in hospitalized patients based on six predictors, including baseline RBC count, neutrophil count ≥7 × 10^9^/L, use of NSAIDs, use of diuretics, alcohol abuse, and previous CKD. Again, these six selected predictors have been shown to be strongly associated with kidney damage in several studies (Kane-Gill and Goldstein, 2015; Motwani et al., 2018; Zhou et al., 2018; Guan et al., 2019; Wang Q. et al., 2019). In addition, we found that in the initial population of this study, D-AKI patients were older than non-D-AKI patients (median 65 vs. 56 years; *p* < 0.001), and advanced age has become a major risk factor for AKI due to changes in renal structure and function in older adults, which has been confirmed and incorporated into many AKI risk models (Mehran et al., 2004; Zhou et al., 2018; Bell et al., 2020). In order to find more targeted D-AKI predictors, we balanced demographic data including age; length of hospitalization and duration of suspected drug exposure by propensity score matching. The predictors we finally found were routinely available in the HIS.

Our study concluded that the reduction in erythrocyte count and neutrophil count ≥7 × 10^9^/L has predictive significance for D-AKI. At present, there are few studies on the relationship between erythrocyte count and AKI, and the mechanism remains unknown. Both inflammation and oxidative stress may play a key role in the progression of AKI (Deswal et al., 2001; Semba et al., 2010). When inflammation occurs, iron metabolism and bone marrow function are affected and the proliferation and maturation of erythrocytes are inhibited (Deswal et al., 2001), resulting in a decrease in erythrocytes counts. In addition, neutrophil count as a marker of inflammation has been shown to provide additional information on the prognosis of AKI (Kane-Gill and Goldstein, 2015; Sun et al., 2018). Therefore, the role of neutrophil count in the prediction of D-AKI should be valued clinically.

In this nomogram model, we found that the combination of non-steroidal anti-inflammatory drugs and diuretics contributed approximately 32 and 45 points to the predicted total score, respectively. Previous studies have shown that the two aforementioned drugs together with angiotensin-converting enzyme constitute a “triple whammy” theory (Loboz and Shenfield, 2005; Dreischulte et al., 2015; Kane-Gill and Goldstein, 2015). The pathological mechanism by which NSAIDs precipitate hemodynamically mediated kidney injury is the inhibition of renal prostaglandins, causing renal vasoconstriction to occur preferentially in the afferent arteries (Khan et al., 2017; Guan et al., 2019). In addition, use of diuretics was found to be a risk factor for D-AKI in many studies (Kane-Gill and Goldstein, 2015; Caspi et al., 2017; Yu et al., 2020). The mechanism is to stimulate the sympathetic nervous system and the renin-angiotensin system (RAS), which leads to hemodynamic changes and eventually renal perfusion deficit leading to AKI (Wang C. et al., 2019). CKD is another important predictor of D-AKI in inpatients. The relationship between CKD and AKI has been mentioned in many studies (Awdishu and Mehta, 2017; Guan et al., 2019; Wang Q. et al., 2019). Recent studies haves shown that both are mutual risk factors and risk factors for cardiovascular disease (Awdishu and Mehta, 2017). Similarly, eGFR, the main diagnostic indicator of CKD, has been used as an independent predictor by multiple AKI prediction models (Bell et al., 2020; Hu et al., 2020). We also found a significant correlation between alcohol abuse and D-AKI. The latest research has pointed out that frequent and occasional binge drinking are associated with a 2.2-fold and 2.0-fold higher risk of CKD progression, respectively, compared with no alcohol consumption (Joo et al., 2020). Hence, clinicians should focus on the renal function of hospitalized patients with a history of chronic kidney disease or alcohol abuse.

The main strength of our study is the first analysis of D-AKI episodes in Chinese hospitalized adult patients by the self-developed ADE-ASAS-2 in a multi-drug environment. We also constructed a D-AKI risk prediction nomogram, which was well identified and calibrated. Compared with the predictors involved in other studies (Zhou et al., 2018; Guan et al., 2019), our model was based on 6 variables that are widely used and easily accessible in clinical practice, and thus be applied in various medical environments.

However, there are several limitations of our study. First, this was a single-center retrospective study and sample selection bias was inevitable. For the same reason, we did not perform external verification. Besides, the construction of this model was based on a case-control study with the risk of observation bias and confounding bias, which was minimized by applying the propensity score matching protocol. Furthermore, since only Scr changes were used to diagnose AKI, this most likely missed some positive cases and underestimated the overall incidence. Finally, some variables that affect the predictive performance of D-AKI, such as cystatin C and interleukin 18 (Connolly et al., 2015; Maiwall et al., 2018), were ignored due to the high percentage of missing values. If these variables are combined, the predictive value may be improved. However, the sample size of this study was insufficient to adequately analyze additional variables, which means that prospective studies with more detailed data and studies with larger sample sizes are needed to verify or update.

## Conclusion

This study provided information on the incidence of D-AKI among adult hospitalized patients and obtained six D-AKI predictors. On this basis, a nomogram was established to predict the occurrence of D-AKI in hospitalized population. This prediction model can help clinicians accurately predict the risk of D-AKI in adult hospitalized patients and identify potential risk patients early. Future studies are still needed for more extensive external validation.

## Data Availability

The original contributions presented in the study are included in the article, further inquiries can be directed to the corresponding author.
